# Direct Interfacial Charge Transfer in All-Polymer Donor–Acceptor Heterojunctions

**DOI:** 10.1021/acs.jpclett.2c02130

**Published:** 2022-09-12

**Authors:** Chenglai Wang, Yuancheng Jing, Liying Chen, Wei Xiong

**Affiliations:** Department of Chemistry and Biochemistry, University of California, San Diego, 9500 Gilman Drive, MC 0358, La Jolla, California 92093-0358, United States; Material Science and Engineering Program, University of California, San Diego, 9500 Gilman Drive, MC 0418, La Jolla, California 92093-0418, United States

## Abstract

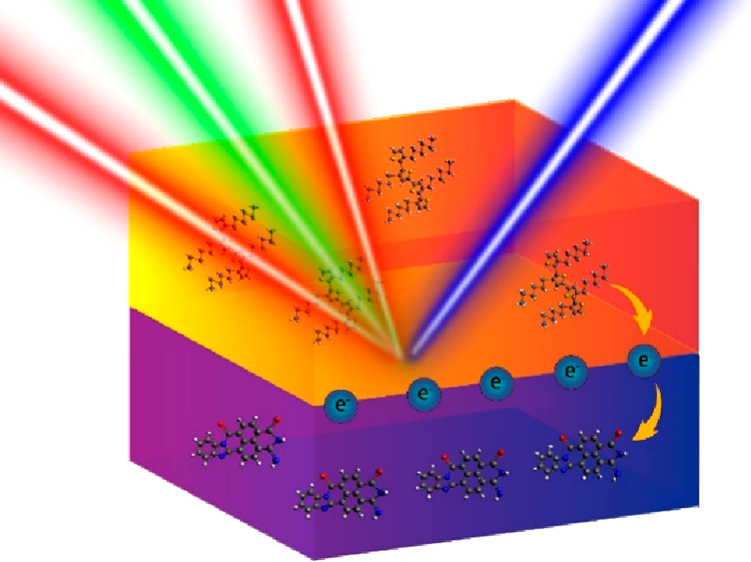

Direct charge transfer at wet-processed organic/organic heterojunction interfaces is observed using femtosecond interfacial sensitive spectroscopy. UV–vis absorption and ultraviolet photoelectron spectroscopy both indicate that a new interfacial energy gap (∼1.2 eV) exists when an interface is formed between regioregular poly(3-hexylthiophene-2,5-diyl) and poly(benzimidazobenzophenanthroline). Resonant pumping at 1.2 eV creates an electric field-induced second-order optical signal, suggesting the existence of a transient electric field due to separated electrons and holes at interfaces, which recombine through a nongeminate process. The fact that direct charge transfer exists at wet-processed organic/organic heterojunctions provides a physical foundation for the previously reported ground-state charge transfer phenomenon. Also, it creates new opportunities to better control charge transfer with preserved momentum and spins at organic material interfaces for spintronic applications.

Direct charge transfer (CT) across interfaces of semiconductor junctions, a phenomenon where charges move from donors to acceptors in one step,^1,2^ provides new opportunities when compared with the common indirect CT in semiconductor materials, where charge transfer involves steps of exciton excitation, migration, and separation.^3−7^ Such opportunities include targeted charge injection to specific sites^8,9^ and preservation of spin states.^10^ However, direct CT is less reported in the literature because it requires sufficient coupling between the energy donors and acceptors.^1,11^ Hence, direct CT has been observed in a limited degree between inorganic materials or interfaces formed from organic and metal materials.^1,3,12,13^ For example, direct CT occurs on two-dimensional transition metal dichalcogenide heterojunctions because of the strong orbital overlap between monolayers.^14,15^ The other examples are direct electron transfer between type II quantum dot materials and plasmonic metal particles^1,16,17^ and between metal electrode and P3HT molecules.^2^ In this case, the evanescent electronic wave functions of donor metals enable strong hybridization with the acceptors.^18^ In contrast, there is only one study on direct CT at organic/organic material interfaces, observed in phthalocyanine–fullerene systems, where direct CT is enabled by well-controlled molecular orientations through a vapor deposition process.^11^

Thus, it is a valid question whether direct CT can occur on wet-processed (e.g., spin-coated) organic semiconductor heterojunctions because, in contrast to the layer deposition methods, wet-processing leads to microscopically less oriented molecules at interfaces.^19,20^ It is uncertain whether such an interfacial molecular arrangement can provide sufficient orbital overlaps between donors and acceptors.

A recent work reported a set of organic photovoltaics (OPVs) using nonfullerene polymers as the n-type acceptor. Through band gap engineering, the authors demonstrated spontaneous ground-state CT at room temperature, which transfers charges without being optically excited to the donor conduction bands.^21^ This thermally excited ground-state CT also implied that direct CT could be possible between organic semiconductor interfaces. The reason is that to enable ground-state CT, the interfacial band gap (energy difference between the HOMO of the donor and the LUMO of the acceptor molecules) needs to be small enough to allow thermal fluctuations to overcome the interfacial energy gap and excite charges from the HOMO of the donor to the LUMO of acceptors, bypassing the large donor band gap. However, direct interfacial CT was not explicitly observed in that study.^21^

In this Letter, using transient vibrational sum frequency generation (VSFG) spectroscopy,^2,13,22^ we show unambiguous evidence of direct interfacial CT on the ultrafast time scale at organic semiconductor junctions, between regioregular poly(3-hexylthiophene-2,5-diyl) (P3HT) and poly(benzimidazobenzophenanthroline) (BBL) (Figure 1a). This implies direct CT is a viable channel between organic semiconductor molecules, and with proper materials engineering, this new pathway could broaden the absorption spectral range of nonfullerene OPVs and explicit control of photocharges at interfaces.

**Figure 1:**
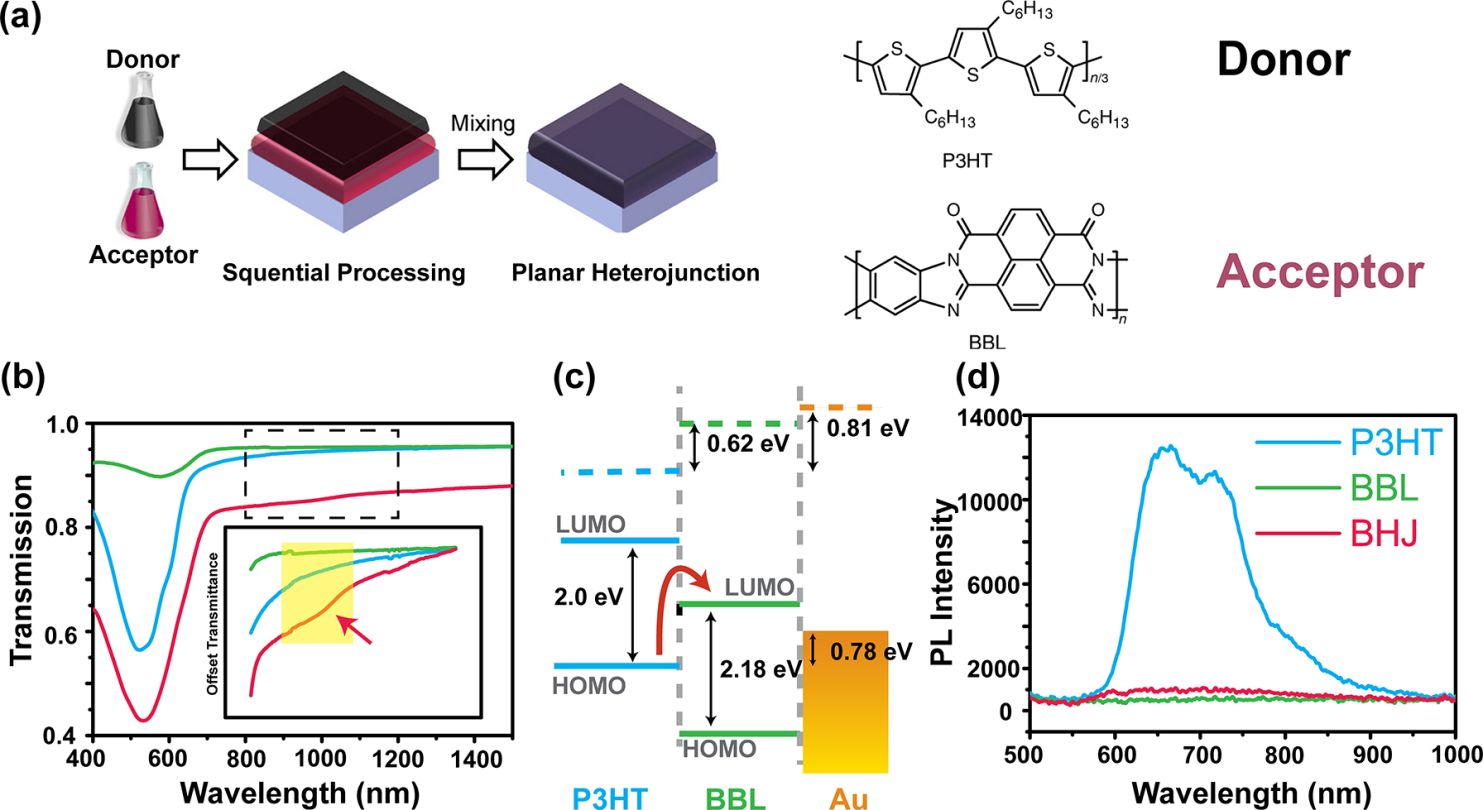
Sample preparation and characterizations. (a) A schematic of preparation for organic planar heterojunction samples. The upper donor layer is P3HT; the lower acceptor layer is BBL. Bilayer films were prepared by spin coating the donor polymers on top of BBL films from orthogonal solvents. (b) UV–vis–near-IR spectra of bulk heterojunction (red), pristine P3HT (blue), and BBL (green) thin films. The absorption peak at around 550 nm is a π–π* transition of the individual polymer. The new optical absorption was obtained by comparing the individual pristine BBL and P3HT absorption spectra with the absorption of P3HT-BBL bilayer. The peak near 1000 nm reveals the CT species could form in the bilayer sample. (c) The energy level diagrams of the all-polymer D–A planar junctions obtained by UPS. The gap between the HOMO of P3HT and the LUMO of BBL is 1.20 ± 0.05 eV. The red arrow indicates the direct CT pathway. (d) Photoluminescent spectra of bulk heterojunction, pristine P3HT, and BBL thin film. Strong photoluminescence quenching is indicative of CT and the formation of a type-II heterojunction. These results suggest that P3HT and BBL orbitals mix with each other well.

We characterized the P3HT/BBL heterojunction using UV–vis–near-IR spectroscopy. A bulk heterojunction (BHJ) was implemented here to maximize the interfacial areas^23^ and thereby to enhance any interfacial transitions. By comparing the spectra of BHJ and the corresponding individual components, we found a broad distinct spectral feature at ∼1000 nm for the BHJ (yellow shaded area in Figure 1b), which was absent in the individual components of BHJ. Thus, this new feature was interface-specific, which could be either a new polaron state or an interfacial CT state.

To further elucidate the origin of this new feature, we performed ultraviolet photoelectron spectroscopy (UPS) to determine the band alignment at polymer interfaces. The sample used was a planar junction (PJ, Figure 1a; details of sample preparation are provided in the Supporting Information), which was necessary to separately determine the HOMO and LUMO levels of P3HT and BBL as well as vacuum level shifts. From UPS data in the low binding energy range (see Figure 2a), we determined the HOMO positions of BBL, P3HT, and PJ relative to the gold Fermi level. From Figure 2b, the vacuum level shift can be characterized by the secondary electron cut off of each polymer and PJ relative to gold. The HOMO positions relative to the materials’ vacuum level can then be determined from the vacuum level shift, HOMO position relative to gold Fermi level, and gold’s work function. We then determined the LUMO from the HOMO position with the band gap of each individual polymer from UV–vis data (2.00 eV for P3HT and 2.18 eV for BBL). Lastly, we determined the energy gap between the HOMO of P3HT and LUMO of BBL by taking their difference and taking into account the vacuum shifts between materials. The interfacial gap was 1.2 eV at the PJ interfaces (Figure 1c; for details, see the Supporting Information and Figure 2). This result agreed with the new NIR absorption feature, indicating this peak was from the interfacial CT state.

**Figure 2:**
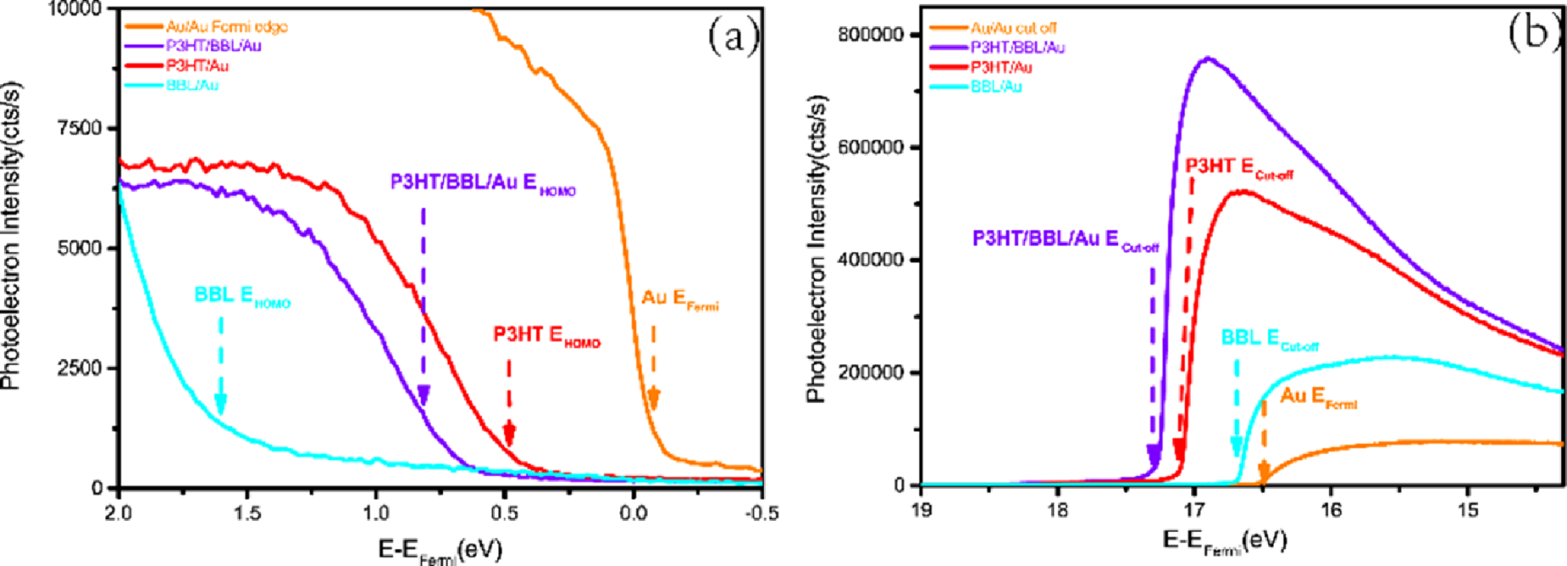
Ultraviolet photoelectron spectroscopy on bare Au, P3HT/Au, BBL/Au, and P3HT/BBL/Au interfaces: (a) UPS spectra near the gold Fermi level region and (b) UPS data near secondary-electron cutoff.

We further performed photoluminescent (PL) measurements on the BHJ samples and compared results with those of the individual components. The PL was collected by exciting the donor materials at 514 nm. From Figure 1d, the pristine P3HT film exhibited a strong PL signal at 700 nm attributed to the recombination of photoinduced electron–hole pairs. After depositing BBL under the P3HT layer, a significant decrease of PL intensity was observed, indicating charge transfer from the LUMO of P3HT to the LUMO of BBL. Therefore, the donor and acceptor have sufficient coupling for indirect interfacial CT. The same coupling could facilitate direct CT as well.^18^

To investigate whether direct CT occurs at wet-processed organic semiconductor interfaces, we applied transient VSFG spectroscopy to the P3HT/BBL PJ (experimental details are in Supporting Information S2). VSFG is a technique that can specifically probe interfaces because of its symmetry selectivity.^24^ A typical VSFG spectrum of the PJ (Figure 3a, right panel) shows a large nonresonant background due to electronic responses to laser excitations. On top of the nonresonant background, there were a few sharp peaks at 2965, 2880, and 2850 cm^–1^. We assigned three peaks as −CH_3_ asymmetric stretch at 2965 cm^–1^, CH_3_ symmetric mode at 2880 cm^–1^, and −CH_2_ symmetric mode at 2850 cm^–1^ from the P3HT alkyl side chain.^25,26^ Because there were three interfaces, air/P3HT, P3HT/BBL, and BBL/SiO_2_, and only the P3HT molecule has −CH_3_ and −CH_2_ features, the resonant VSFG signal could come only from the air/P3HT and P3HT/BBL interfaces. When studying the charge transfer dynamics, we observed only a transient signal from the P3HT/BBL interface, because the air/P3HT interface showed no transient signal upon optical pumping (see Figure S4 for the null signal from P3HT on glass sample, which comprise air/P3HT and P3HT/glass interfaces).

**Figure 3:**
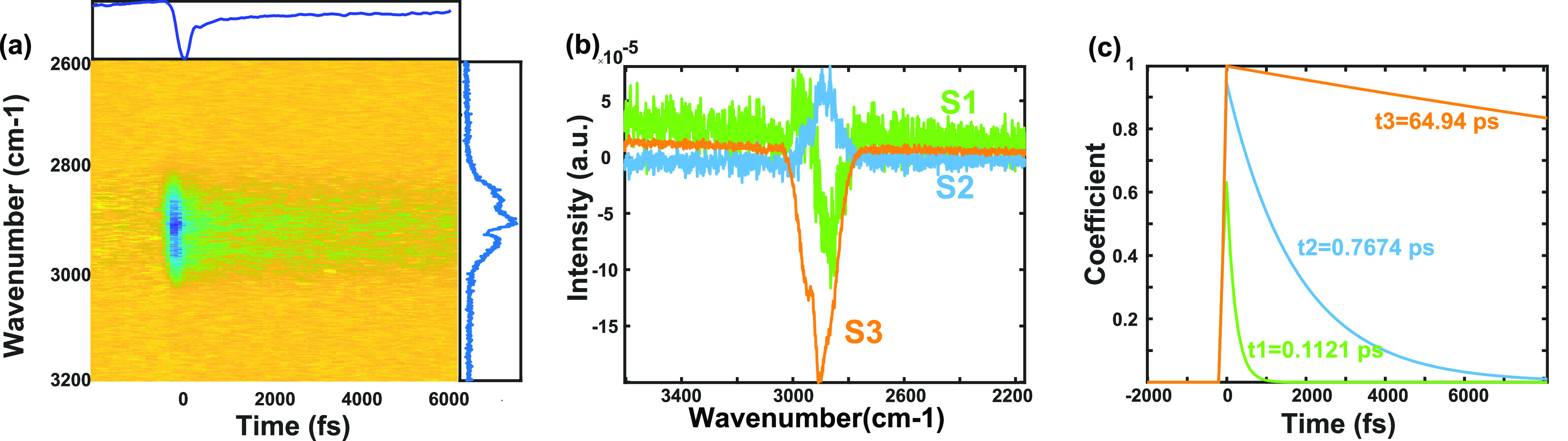
Tr-VSFG spectroscopy and analysis result on P3HT/BBL interface. (a) Pseudo color 2D tr-VSFG spectrum. A negative signal is observed at *t* = 0, which recovers quickly and remains as a bleach signal at *t* > 0. Top, integrated tr-VSFG signal; right, linear VSFG spectrum. Global analysis results: (b) spectral and (c) dynamic components. S1 is coherent artifact originating from laser response. S2 and S3 are two simultaneous charge dynamics.

If CT occurs when a pump pulse centered at 1030 nm is applied to the PJ, the separated electrons and holes could generate an electric field at the interfaces. The new electric field triggers additional third-order nonlinear signals that modulate the VSFG signals, known as the electric-field induced (EFI) effect.^27,28^ The EFI signals have been demonstrated in second harmonic generation and VSFG in both external and internal electric field settings as well as transient electric field through ultrafast charge transfer.^2,7,11,27,28^

The transient VSFG spectrum of the PJ clearly showed a time-dependent spectral change (Figure 3a). The spectrum had a negative peak around time zero, and then transient signals recovered to a negative amplitude with a long lifetime. On the basis of global analysis results, we found that the spectral dynamics could be decomposed into three parallel components (Figure 3b,c), which are similar to the P3HT/metal system.^13^ Components 1, 2, and 3 have lifetimes of ∼110 fs, 770 fs, and >60 ps, respectively. Component 1’s time scale agreed reasonably with the instrumentation response (determined from Au photoresponses, Figure S4c), so it originated from the coherent artifacts;^29^ component 2 decayed to zero, which indicated a charge recombination pathway; component 3 had a very long relaxation lifetime, which agreed with the direct CT across the P3HT/Au interface.^2,13^ Thus, the long-lifetime component 3 could be a signature of the direct CT at organic/organic heterojunction interface, which recombines later. The rise dynamics of the direct CT is beyond the instrumental response. The recombination lifetime agrees with the lower limit of lifetime in the literature reasonably well, and the differences could stem from the short scanning range of our experiments or the different nonfullerene materials.^30,31^ However, the dynamics were dominated by the nonresonant signal, whereas the vibrational features were not apparent in the dynamics. Thus, we analyzed the dynamics based on the integrated signal intensity, like a transient SHG measurement.

We further performed additional experiments to examine direct interfacial CT. First, we measured the transient VSFG signal intensity as a function of pump fluence. The transient VSFG intensity was linearly dependent on the pump fluence, which indicated that below 5 mW pump fluence, the dynamics were triggered by single-photon absorption; that is, two-photon absorption does not occur (Figure 4a). Above 5 mW, the transient signal started to saturate and then decrease, indicating sample degradation (Figure S5). Second, when pumped with 1500 nm (∼0.8 eV) pulses, under the same pump fluence, there was no obvious photoinduced dynamics. Thus, 0.8 eV was not high enough to initiate the CT process (Figure 4b). These results were consistent with the 1.2 eV interfacial band gap determined from UPS, suggesting that the 1030 nm (1.2 eV) pump pulse lifted electrons from HOMO of P3HT directly to the LUMO of BBL through a one-photon absorption process. In addition, no transient VSFG signals were observed in donor-only and acceptor-only samples (Figure S4a).

**Figure 4:**
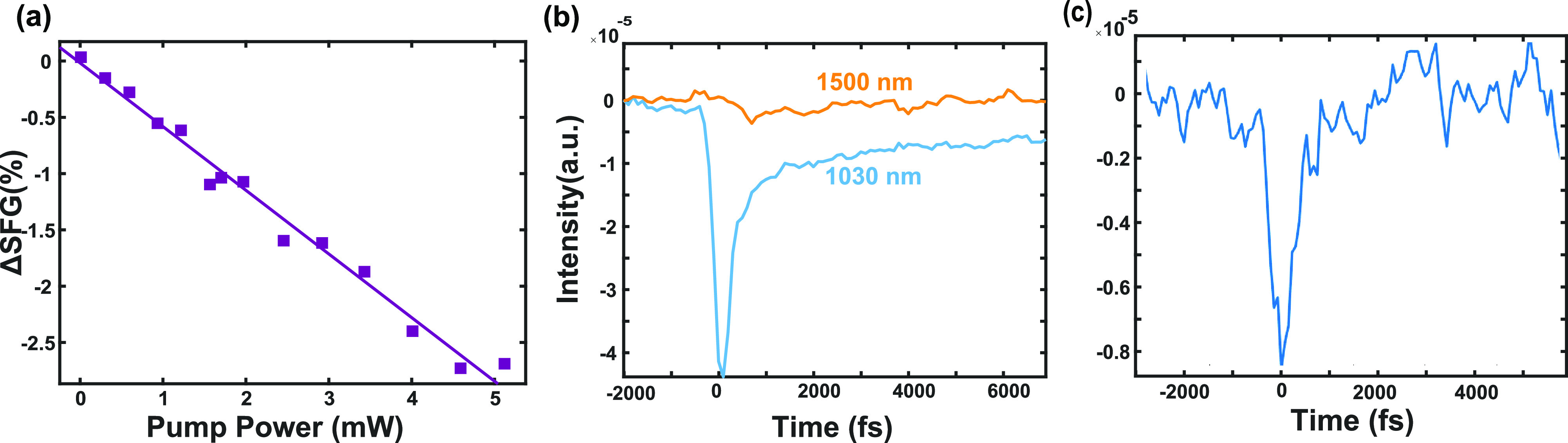
Dynamics studies to reveal charge transfer mechanisms. (a) Power dependence on Tr-VSFG dynamics. The Tr-VSFG signal linearly depends on the pump power. (b) Dynamics of P3HT/BBL pumped by a 0.8 eV (1500 nm) pulse. No dynamics were observed, in contrast with the result when pumped with 1030 nm pulse. (c) Tr-VSFG signal of polaron samples showed only a fast decay dynamic, different from the signal of PJ.

We last compared the direct interfacial CT dynamics with polaron formation dynamics. We measured a small-molecule doped polaron sample: PMA-doped P3HT, which had a similar NIR spectral feature at 980 nm.^32^ Its transient VSFG dynamic follows a delta function at *t* = 0, similar to coherent artifacts, with no signal at long time delays (Figure 4c). This result suggested that when polarons are excited, there is no electric field generated across the interface and no corresponding long-lasting transient EFI-VSFG signals.

To shed light on the mechanisms of charge recombination after the direct interfacial CT, we measured the dynamics at different power (Figure 5a). We found the lifetime of the direct recombination dynamics to be power-independent (Figure 5b), while the decaying dynamics of the signals due to charge separations became faster as the pump power increased (Figure 5c). Thus, the direct recombination channel was a germinate process—the CT pairs did not dissociate,^33,34^ whereas the power dependence of the charge-separated channel suggested that free charge carriers recombined through a nongerminate process, depending on the carrier density.^35−37^ Since charges were directly transferred across interfaces, they could either directly recombine or separate. This suggests strong Coulomb interactions between charges are still significant in electrons and holes across interfaces which could prevent charge separations.^33,38−40^

**Figure 5:**
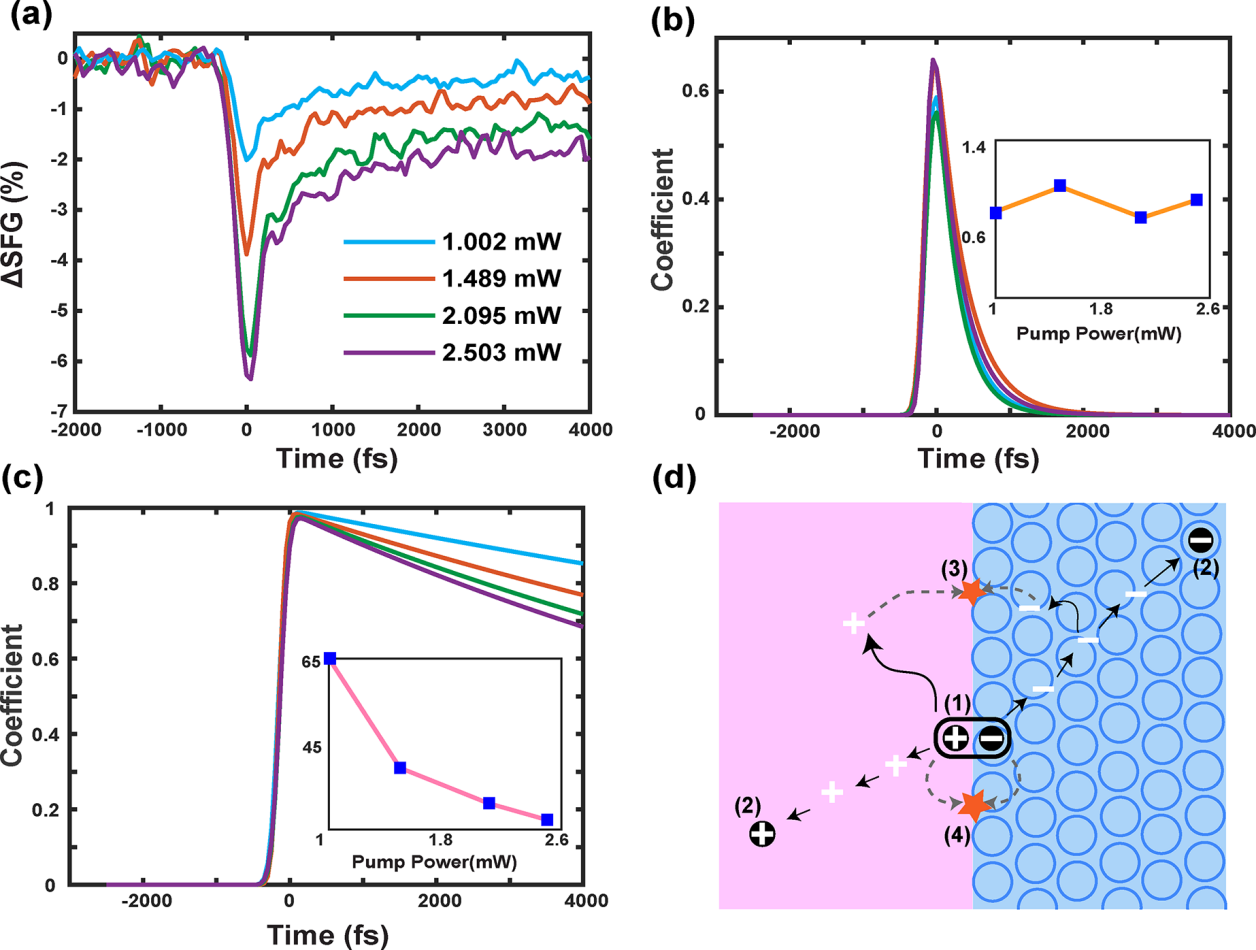
Dynamics under various pump fluences. (a) Tr-VSFG dynamic traces of P3HT/BBL interface at various pump fluences. (b and c) Global analysis results of panel a. The dynamics with different fluences are separated into two simultaneous pathways. (b) The dynamics of S2, which represents the fast recombination dynamics, was a germinate process since there is no dependence of lifetime on the pump fluence. (c) The recombination mechanism of S3, the separated interfacial charges, was a nongerminate process because it inversely depends on the pump fluence. Inset: lifetime (picosecond) versus pump fluence (mW). (d) Schematic of the interfacial charge dynamics: (1) Photon absorption creates charge transfer pairs directly at interface. (2) A fraction creates separated charges and (3) recombination through a nongeminate process. (4) Other interfacial electron hole pairs quickly recombine through a geminate process.

We conclude the near-IR excitation can directly promote electrons from the donor (P3HT) to the acceptor (BBL) molecules across interfaces of solution phase-processed organic junctions (step 1 in Figure 5d). Only a fraction of the direct CT electron–hole pairs separates into free charge carriers (step 2, incident photon to charge efficiency is ∼10^–5^; see Supporting Information S7), whereas the remainder exist as bound CT complexes. The former can diffuse and later recombine in a nongerminate way (step 3), whereas the latter reaches the ground state by geminate recombination (step 4).

This finding extends the direct interfacial CT previously reported in only inorganic or metal junctions^14,41−43^ to organic heterojunctions. Considering that P3HT and BBL are both typical semiconductor polymers and that the samples are prepared by a regular wet process, such as solution phase spin coating, the present work indicates that even under solution phase processing, the overlap between donor and acceptor molecules could be sufficient to enable direct CT. Specifically, the π–π interactions of the conjugated groups in both donor and acceptor molecules could facilitate self-organizations between them and maximize the interactions for CT to occur.^44−46^ It remains to be further verified how extensive direct interfacial CT in organic semiconductor heterojunctions is.

Because direct interfacial CT is always below the band gap of individual components, the interfacial band gap likely falls into the near-IR regime, which could extend the spectral range of OPV from the visible to the near-IR. Furthermore, because direct interfacial CT is a one-step process, it would be critical for preserving momentum and spin characteristics during the transfer process, which is important in spin transfer applications.

## Supporting Information Available

Additional discussion of sample preparation, transient VSFG spectrometry, UPS measurement, and incident photon-to-charge efficiency (PDF)
Transparent Peer Review report available (PDF)

## Notes

All data and codes used to plot the data can be found at https://github.com/EthanJing1/Data_ChargeTransfer.git.
