# Supplementary material for: Direct Interfacial Charge Transfer in All-Polymer Donor–Acceptor Heterojunctions
Source: J Phys Chem Lett. 2022 Sep 12;13(37):8733–9. doi: 10.1021/acs.jpclett.2c02130 (PMC9511559; doi:10.1021/acs.jpclett.2c02130)
Supplement: Supplementary file 1 — jz2c02130_si_001.pdf [file jz2c02130_si_001.pdf]

Supporting Information

Direct Interfacial Charge Transfer in All-Polymer Donor-Acceptor Heterojunctions

Chenglai Wang, Yuancheng Jing, Liying Chen, Wei Xiong

Department of Chemistry and Biochemistry, University of California, San Diego, 9500 Gilman Drive, MC 0358, La Jolla, California 92093-0358, United States

Material Science and Engineering Program, University of California, San Diego, 9500 Gilman Drive, MC 0418, La Jolla, California 92093-0418, United States

AUTHOR INFORMATION

Corresponding Author

*E-mail: w2xiong@ucsd.edu.

S1. Sample preparation:

The P3HT/BBL system is prepared through a spin coating method. Prior to use, the glass substrates were sonicated in acetone for 15 min followed by ozone cleaning for 20 min. High-molecular-weight BBL was dissolved in MSA at room temperature at a concentration of 2 mg/ml. BBL films were fabricated by spin coating on glass at 3,000 r.p.m. for 1 min, immediately followed by immersing into (1) isopropanol and (2) deionized water to remove any residual MSA from the film. Regioregular P3HT polymers (REIKE metals) are dissolved in chloroform to obtain a 5 mg/ml solution, which is then spin coated onto the glass substrates using 3,000 r.p.m. spin speed.

S2. Transient VSFG spectrometer

The transient vibrational sum frequency generation (tr-VSFG) spectrometer utilizes a pump probe geometry. A high-repetition-rate femtosecond laser system (Pharos, 100kHz, Light Conversion) was used for the transient VSFG measurement. The Pharos laser generates 160 fs pulses centered at 1030nm with a pulse energy of 100 μJ. The 1030nm pulses are separated into two parts. The major part is used to pump a high power optical parametric amplifier (Orpheus-HP, Light Conversion) to produce tunable IR pulses. The residual 1030nm after OPA is used to prepare an upconversion pulse by passing through an etalon filter (SLS optics) to generate narrow-band pulse with a bandwidth of 7 cm^{-1} at FWHM. The minor part of 1030nm fundamental pulse serves as the pump beam, followed by passing through a variable-length delay line before arriving at the sample. The power of the pump beam is controlled by a waveplate and polarizer pair. The VSFG signal serves as the probe which is generated by overlapping the mid-IR beam and the 1030 nm upconversion beam both spatially and temporally at the sample surface. The center wavelength of the mid-IR is tuned to 3.5 μm, on resonance with the C-H vibrational modes of P3HT. The signal is collected by another parabolic mirror, collimated, and send to the spectrograph and CCD detector. In this work, a repetition rate of 10 kHz was used to avoid sample damage. The mid-IR beam and the 1030 nm beam are focused by a f=10 cm parabolic mirror onto the sample to generate the VSFG signal. The angles of incidence are 45° for IR and 60° for near-IR beams. All SFG measurements were carried out under PPP polarization (p-SFG, P-visible, p-IR). SFG signal was dispersed by a spectrograph (Shamrock, Andor) and detected by a charge coupled device (Newton idus, Andor).

All samples are loaded in an air-tight cell and purged with nitrogen gas to eliminate sample burning issues. Each scan has at least 3 averages and between each average the sample is rasterred to a different spot. A shutter in the pump beam line is used to modulate the pump beam on and off. The time delay between pump and prove pulses is randomized within the region of interest.

ΔSFG is calculated using the following equations:

$$I_{pumpon} = \left|\chi^{(2)} E_{IR} E_{vis} + \chi^{(3)} E_{IR} E_{vis} E_{DC}\right|^2. \quad \text{(Eq. 1)}$$

$$I_{pumpoff} = \left|\chi^{(2)} E_{IR} E_{vis}\right|^2. \quad \text{(Eq. 2)}$$

$$\Delta SFG(t) = \frac{I_{pumpon} - I_{pumpoff}}{I_{pumpoff}} \propto \frac{2Real(\chi^{(3)} E_{IR} E_{vis} E_{DC}(t))}{|\chi^{(2)} E_{IR} E_{vis}|}. \quad \text{(Eq. 3)}$$

S3. UPS measurement

Sample preparation:

Ultraviolet photoelectron spectroscopy (UPS) was applied on bare Au, P3HT/Au, BBL/Au and P3HT/BBL/Au samples for investigating the band alignment at P3HT/BBL interface. Bare Au served as reference and substrates for the measurement. The information of HOMO energy level and vacuum energy level of P3HT, BBL can be revealed by UPS data[1][2]. Figure S1 shows the fundamental mechanism of UPS. The valence electrons of surface material are excited by absorbing the energy of UV photon from Helium (hv=21.2eV) and ionized at certain kinetic energy. An electron energy analyzer is used for detecting the kinetic energy and distribution of ionized electrons and to reveal the density of states of occupied molecular orbital. Electrons in the top of valence band have the lowest binding energy and therefore will be the easiest to be ionized with the highest kinetic energy. UPS spectra below showed the distribution of ionized electrons at different binding energy.

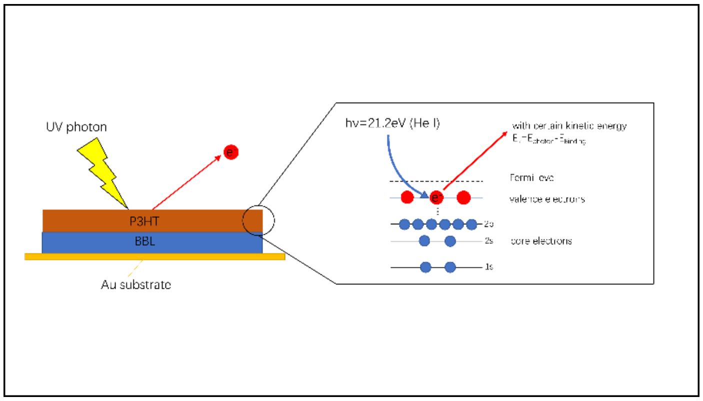

Fig. S1. Schematic working mechanism of UPS.

Figure S2 shows the energy relationship in UPS of BBL sample. The following steps can be taken for extracting band information from UPS spectra:

1. Calculating the energy difference (ε_FHOMO) between HOMO edge of BBL and Fermi level of Au:
$$\varepsilon_F\text{HOMO} = E_{HOMO(BBL)} - E_{Fermi(Au)} = 1.76eV - 0eV = 1.76eV \quad \text{(Eq. 4)}$$
2. Calculating the energy difference ($-\Delta$) between secondary electron cutoff of BBL and Au, this energy difference equals the vacuum level shift between BBL and Au:
$$-\Delta = E_{Cut-off(BBL)} - E_{cut-off(Au)} = 16.68eV - 16.49eV = 0.19eV \quad \text{(Eq. 5)}$$
3. Calculating the work function of Au (Φ_{Au}):
$$\Phi_{Au} = hv - E_{cut-off(Au)} = 21.2eV - 16.49eV = 4.71eV \quad \text{(Eq.6)}$$
4. Calculating the energy difference between vacuum level and HOMO position of

BBL (εHOMO):

$$\varepsilon\text{HOMO} = \varepsilon_{\text{F}}\text{HOMO} + \Phi_{Au} + \Delta = 1.76eV + 4.71eV + (-0.19eV) = 6.28eV$$

5. Calculating the position of LUMO (requiring UV-vis data):

$$E_{LUMO} = E_{\text{HOMO}} + \text{band gap}(2.18\text{eV}) \quad \text{(Eq.7)}$$

Then the band structure of BBL is obtained. Similarly, from Figure S3, the band structures of the P3HT and P3HT/BBL/Au samples can be obtained. The results are summarized in Table S1.

	E_{HOMO}	$E_{cut-off}$	$\varepsilon_{\text{F}}\text{HOMO}$	$-\Delta$	εHOMO	$Bandgap$
Au	0eV	16.49eV	--	--	--	--
BBL/Au	1.76eV	16.68eV	1.76eV	0.19eV	6.28eV	2.18eV
P3HT/Au	0.54eV	17.12eV	0.54eV	0.63eV	4.62eV	2.00eV
P3HT/BBL/Au	0.78eV	17.30eV	0.78eV	0.81eV	4.68eV	2.00eV

Table S1. UPS summarized data

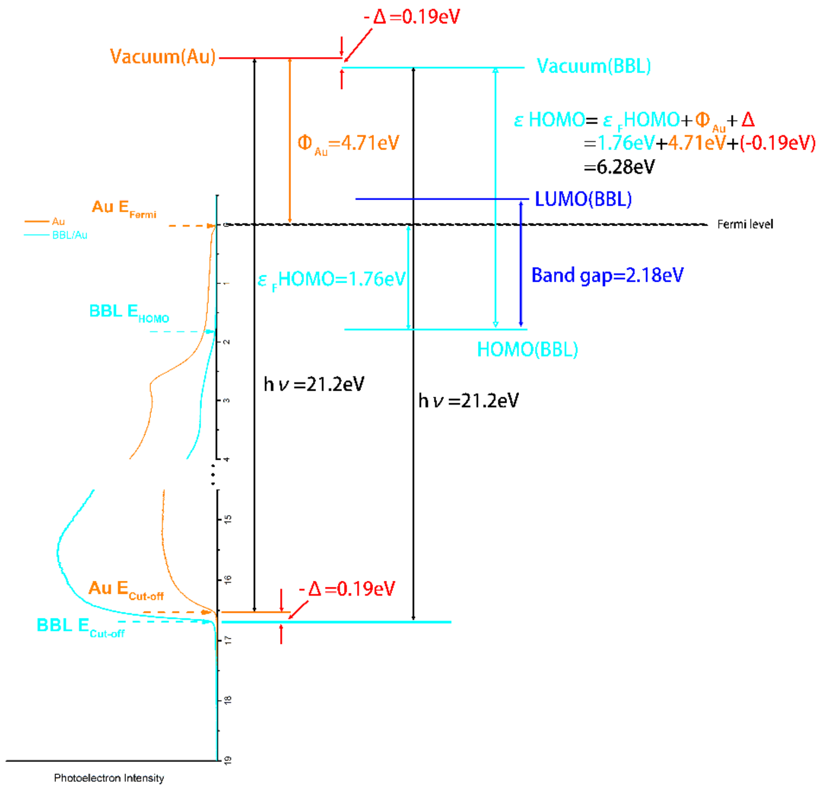

Fig. S2. Energy relationship in UPS

From Table S1, The HOMO position and vacuum energy level both have shifted between P3HT and P3HT in the heterojunction indicating charge transfer at the polymer interface. The band alignment of P3HT/BBL/Au is obtained and summarized in fig S3. The interfacial band gap is determined by $E_g = HOMO(P3HT) - LUMO(BBL) + \Delta = 4.68eV - 4.10eV + 0.62eV = 1.20eV$

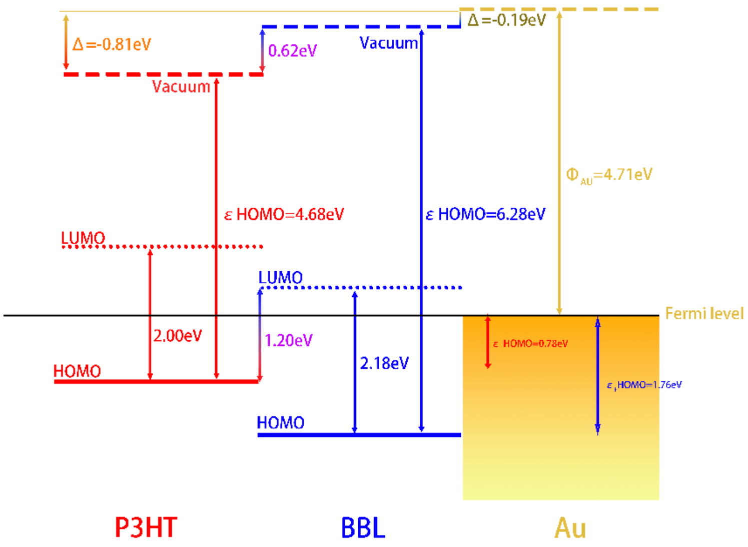

Fig. S3. Band alignment of P3HT/BBL/Au interfaces

We note that in Ref 22, an interfacial band gap of 0.57 eV was reported for the same materials. The gap reported there was before considering vacuum level shift, and when it is included, the interfacial gap is 1.01 eV, which agrees with our measurement within the error bars.

S4. Supplementary figures and tables

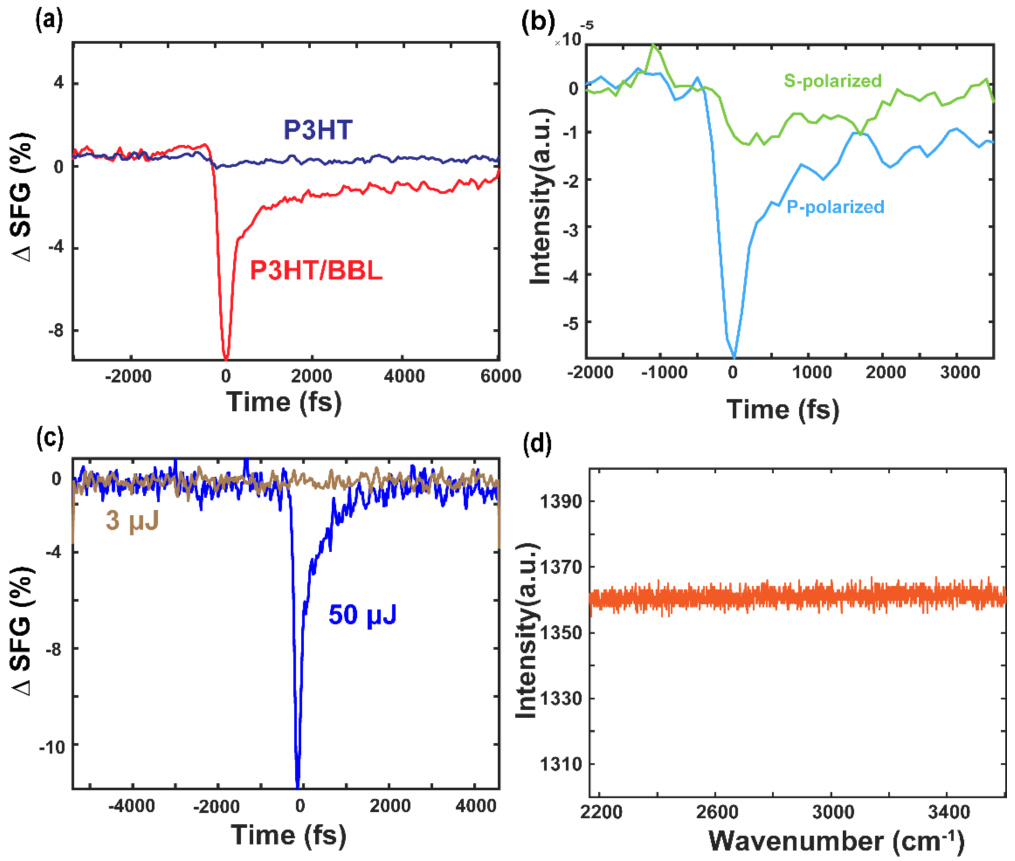

Figure S4. tr-VSFG dynamics of the control experiments (a) No dynamics is observed at only P3HT/glass interface (blue) while under same pump fluence. P3HT/BBL interface (red) shows a dynamic feature. This result shows no thermal effect is modulating the absorption cross section that can generate a fake dynamic trace. No static VSFG signal is observed at BBL/glass interface. Therefore, we didn't measure the time-resolved spectrum for BBL/glass interface. (b) Pump polarization dependence of the tr-VSFG signal. The transient signal is significantly reduced when using S polarized pump, indicating the charge transfer is dominated by a dipole driven process, perpendicular to the interfaces. (c) Bare gold surface shows a spike at time zero under high pump power (blue) which is assign to coherent artifact, whereas no dynamics shows up under low pump power (grey). The lifetime of coherent artifact is 150fs. (d) No SFG response on BHJ sample shows that the molecules are randomly oriented at the interfaces (1360 is the background level of the CCD).

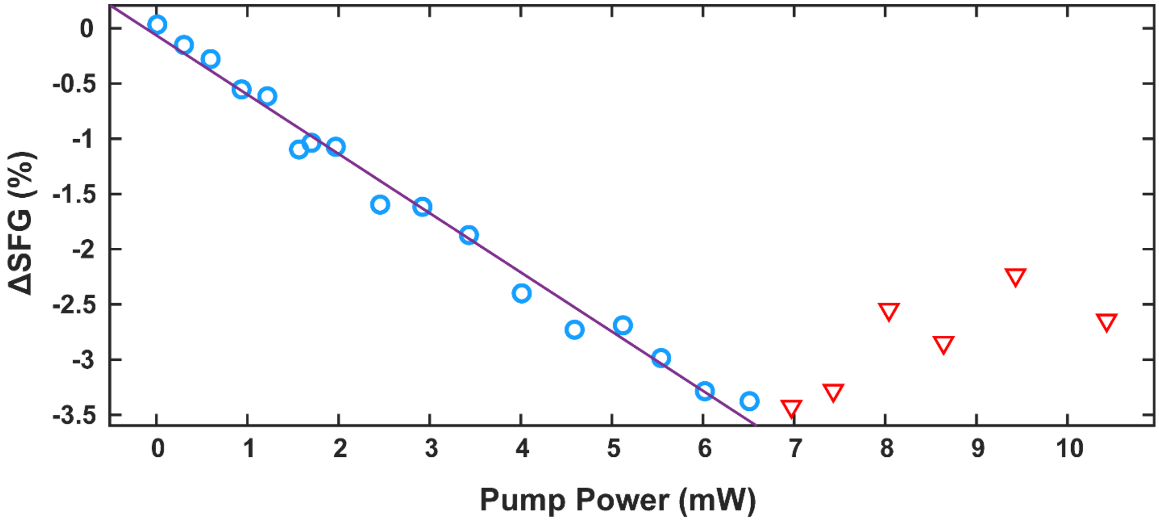

Figure S5. Power dependence on Tr-VSFG intensity. The signal intensity linearly depend on the pump power until above 7mW, where the signal (red triangles) first saturates in intensity and then decreases, indicating at this power level, the sample might be photodegraded.

S5. SEM cross section image

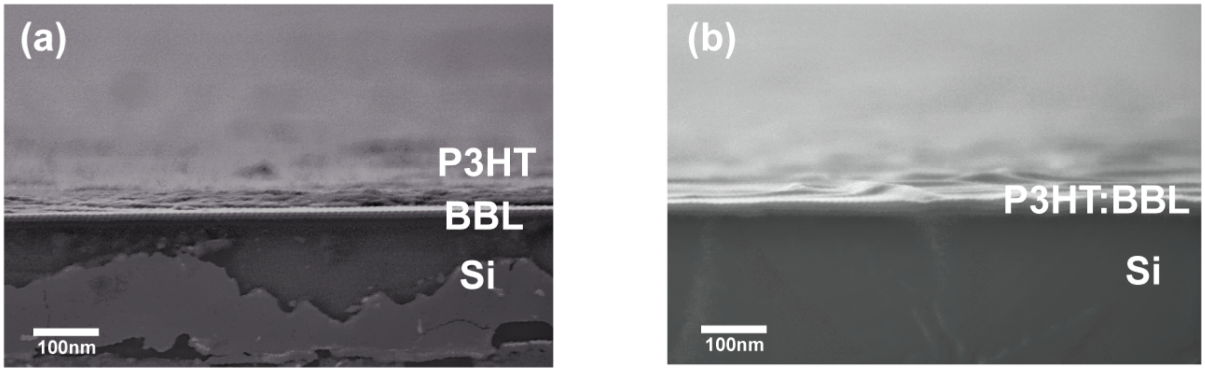

Figure S6. SEM cross section image showing the P3HT/BBL bilayer spin-coated on Si wafer (a) P3HT/BBL bilayer structure, double layer structure suggests planar junction formation. The acceptor P3HT layer is about 50nm, the donor BBL layer is about 20nm. (b) Bulk heterojunction, only one layer is resolved.

To further confirm the planar junction geometry, we conducted SEM measurement (Figure S6 left) and the P3HT and BBL layers can be distinguished, suggesting planar junction formation. The SEM cross section image (Figure S6, right) confirmed that in BHJ, the donors and acceptors are mixed in one-layer in bulk heterojunction. When there are randomly oriented molecules at the interfaces, such as BHJ, there is no VSFG response. This result can be understood as in BHJ, even charge transfer occurs and generate an DC electric field, this DC field is isotropic, so the net DC field is still zero, which will not generate additional VSFG signal through the E-field induced $\chi^{(3)}$ VSFG process.

S7. Incident photon to charge efficiency

Transient VSFG intensity can be used to determine the incident photon to separated charge conversion efficiency. The number of free charge carriers across interfaces is proportional to the strength of interfacial electric field, on which the transient VSFG intensity is linearly depended, based on Eq.8 (assuming P3HT/BBL as a parallel capacitor),

$$E = \frac{1}{\varepsilon_r \varepsilon_0} \cdot \frac{Q*e}{A} \quad \text{(Eq.8)}$$

Where Q is the total number of photoinduced charge at interface, A is irradiative area of 1030nm pump beam ($0.0249 mm^{-2}$), ε_r is dielectric constant of P3HT/BBL system (about 2.5), ε_0 is the permittivity of vacuum ($8.\ 854187817 \times 10^{-12}$ C/(V*m)), and e is 1.6×10^{-19} C/electron.

From our data, when the pump fluence is 2.5mW, the ΔSFG is about 2.5%. Based on Massari's work (27), the same amount SFG modulation was observed at an external potential U of 0.66V, across a layer of 150 nm thickness. Thus, the corresponding interfacial electric field E=U/d= $4.4 \times 10^6\ V/m$.

The charge density (Q/A) separated at interface is given by:

$$Charge\ density = \frac{Q}{A} = E \cdot \varepsilon_r \varepsilon_0 / e = \frac{U}{d} \cdot \varepsilon_r \varepsilon_0 / e = 6.08 \times 10^{14}\ m^{-2} \quad \text{(Eq.9)}$$

Incident photon flux is calculated based on the measured pump (0.22 μJ) and photon energy (1.2 eV)

$$Photon\ flux = \frac{pump\ energy}{photon\ energy} \cdot \frac{1}{A} = \frac{0.25 \times 10^{-6} J}{1.92 \times 10^{-19} J} \cdot \frac{1}{0.0249\ mm^2} = 5.23 \times 10^{19}\ m^{-2} \quad \text{(Eq.10)}$$

Therefore, the conversion efficiency from incident photon flux to free charge carrier density is:

$$\eta = 1.2 \times 10^{-5}$$

Reference

[1]. Braun, S., Salaneck, W.R. and Fahlman, M., 2009. Energy-level alignment at organic/metal and organic/organic interfaces. *Advanced materials*, *21*(14-15), pp.1450-1472.

[2]. Xiang, B., Li, Y., Pham, C.H., Paesani, F. and Xiong, W., 2017. Ultrafast direct electron transfer at organic semiconductor and metal interfaces. *Science advances*, *3*(11), p.e1701508.
