# Supplementary material for: Direct Interfacial Charge Transfer in All-Polymer Donor–Acceptor Heterojunctions
Source: J Phys Chem Lett. 2022 Sep 12;13(37):8733–9. doi: 10.1021/acs.jpclett.2c02130 (PMC9511559; doi:10.1021/acs.jpclett.2c02130)
Supplement: Supplementary file 2 — jz2c02130_si_002.pdf [file jz2c02130_si_002.pdf]

jz-2022-02130r.R1

Name: Peer Review Information for "Direct Interfacial Charge Transfer in All-Polymer Donor-Acceptor Heterojunctions"

First Round of Reviewer Comments

Reviewer: 1

Comments to the Author

1. What is the major advance reported in the paper?

This is a wonderful work from Dr. Xiong group. In this work, the authors reported direct charge transfer at wet-processed organic/organic heterojunction using femtosecond transient vibrational sum frequency generation (VSFG) spectroscopy. They found that an electric field-induced second-order optical signal was established after resonant pumping. The attributed it to be the existent of a transient electric field due to separated electrons and holes at interfaces, followed by non-geminate recombination.

2. What is the immediate significance of this advance?

This work opens an avenue of revealing charge transfer occurring at interfaces. It provides a physical foundation for the previously reported ground state charge transfer phenomenon and also creates new opportunities to better control charge transfer with preserved momentum and spins at organic material interfaces for spintronic applications.

3. Technical suggestions

1. Please justify why “non-geminate” recombination was assigned;
2. Instrumental response time in the kinetic traces should be added;
3. In Fig. 4, a larger range of the powers used should be checked;
4. Did the authors use different polarization to do the experiments? Which polarization was used for the demonstrated results?

Reviewer: 2

Comments to the Author

The manuscript by Wang et al. examines the existence of direct interfacial charge transfer (CT) in an all-polymer donor-acceptor heterojunction via transient VSFG and photoelectron spectroscopy. Direct CT could potentially improve the performance of organic electronic devices without using dopants, but it has only been observed in one previous study, and its dynamics and mechanism are largely unknown. Hence, this study is an important step towards a better understanding of CT in organic semiconductors. I would support its publication after the authors addressed the following comments:

- It would be better to clarify the following terms used in the manuscript: multi-step CT, direct CT, (spontaneous) ground-state CT, photo-induced direct interfacial CT, and indirect CT. It's helpful for the readers to know the differences between them. I would suggest the authors add "photo-induced" to the title to make things clear.

- Line 27-32 on page 6: "Therefore, the donor and acceptor had sufficient orbital mixing for indirect interfacial charge transfer to occur, a prerequisite for direct charge transfer." I don't understand this conclusion. Why is orbital mixing for indirect CT a prerequisite for direct CT?

- The energy level diagram in Fig. 1 is different from that in Ref. 21, although both looked at P3HT/BBL. The energy gap between P3HT HOMO and BBL LUMO is 1.2 eV in this work, but is about 0.6 eV in Ref. 21. Any explanation for this difference?

- I wonder if it is possible to estimate the fractions of the photo-induced direct CT pairs that separate into free charges, undergo germinate and non-germinate recombinations (i.e., processes (2), (3), and (4) in Fig. 4(d)) from their transient VSFG.

- There are some typos in the manuscript (not an exhaustive list): for example, line 15 on page 2, should be "the existence of"; line 22 on page 4, should be "photo-induced direct interfacial charge transfer"

Reviewer: 3

Comments to the Author

This work characterized direct interfacial charge transfer at the heterojunction between two polymer materials. The charge transfer discovered in wet-processed polymer donor/acceptor interface will provide new platform for both fundamental study and device applications of organic photovoltaics. I would suggest publication in the journal of physical chemistry letters, after addressing the following few questions:

1. Would there be any PL signature that may correspond to the radiative recombination of the separated charges at the interface, especially at around 1000 nm similar as the absorption?
2. What is the polarization of the lasers used for SFG pump-probe experiment? Is it mostly in-plane or out-of-plane?
3. Are there any current or past time-resolved PL or transient absorption measurements for similar polymer interfaces that shows a comparable lifetime of charge carrier transfer state?
4. Could the rate of initial fast charge transfer resolved in the SFG dynamics at times close to time zero?
5. What is the polymer thickness prepared from the spin coating process? What would happen if the solvent flow in the spin coating process mix the two polymers, leading to randomly oriented molecules at the interfaces? In that case, would there still be an SFG response if they are homogeneously mixed? Maybe a cross section characterization of the polymer interface would be helpful to illustrate a clear planar heterojunction instead of random mixing.
6. The UPS measurement of the orbital energies is a crucial information in determining the HOMO LUMO offset. It will be helpful to include the UPS data in a main text figure instead of the supplementary.
7. Would there be any expansions for the negative sign of VSFG of component 3 and positive sign of component 2?

Author's Response to Peer Review Comments:

Dear Editor,

We thank both Reviewers for their comments and suggestions that help us strengthen our manuscript in both scientific rigor and broader readership. We have addressed all Reviewers' comments point-by-point and revised the manuscript accordingly.

Reviewer: 1

1. What is the major advance reported in the paper?

This is a wonderful work from Dr. Xiong group. In this work, the authors reported direct charge transfer at wet-processed organic/organic heterojunction using femtosecond transient vibrational sum frequency generation (VSFG) spectroscopy. They found that an electric field-induced second-order optical signal was established after resonant pumping. The attributed it to be the existent of a transient electric field due to separated electrons and holes at interfaces, followed by non-geminate recombination.

2. What is the immediate significance of this advance?

This work opens an avenue of revealing charge transfer occurring at interfaces. It provides a physical foundation for the previously reported ground state charge transfer phenomenon and also creates new opportunities to better control charge transfer with preserved momentum and spins at organic material interfaces for spintronic applications.

Reply: We thank the reviewer's positive assessment to our work.

3. Technical suggestions

Q1. Please justify why "non-geminate" recombination was assigned.

Reply: We thank the Reviewer for pointing out the confusion. Here, we consider that the long lifetime in the planar heterojunction could arise from two possibilities: (1) a nongeminate recombination of free charge carriers; (2) a geminate recombination of trapped states. To differentiate them, we performed a pump fluence-dependent experiment (Fig.4), which showed that the slow recombination dynamics is a non-first order process. Thus, we assigned the recombination to be non-geminate, as a geminate CT recombination should follow first order kinetics.

2. Instrumental response time in the kinetic traces should be added;

Reply: We thank the Reviewer for raising that issue. We have included instrumental response time in supplemental materials. The instrumental response was determined by performing pump probe experiment on bare gold surface (as shown in Fig. S5c). The signal give the instrument response to be 150 fs.

3. In Fig. 4, a larger range of the powers used should be checked;

Reply: We thank the Reviewer for the suggestion. We performed a higher pump fluence experiment as the Reviewer suggested. As shown in Fig.R1, the Tr-VSFG signal linearly depends on the pump power in a larger range. When the pump fluence higher than 7mW, the signal first was saturated and then decreased, possibly due to sample degradation. This result suggests that the pump fluence used in our experiment was well within the single photon absorption limit, and sample degradation was mitigated.

We have included this figure as Figure S6, and added a statement in the main manuscript “below 5 mW pump fluence, the dynamics were triggered by single photon absorption, i.e. no two photon absorption occurs (Fig.3a). Above 5mW, the transient signal started to saturate and then decrease, indicating sample degradation. (Figure S6.) ” .

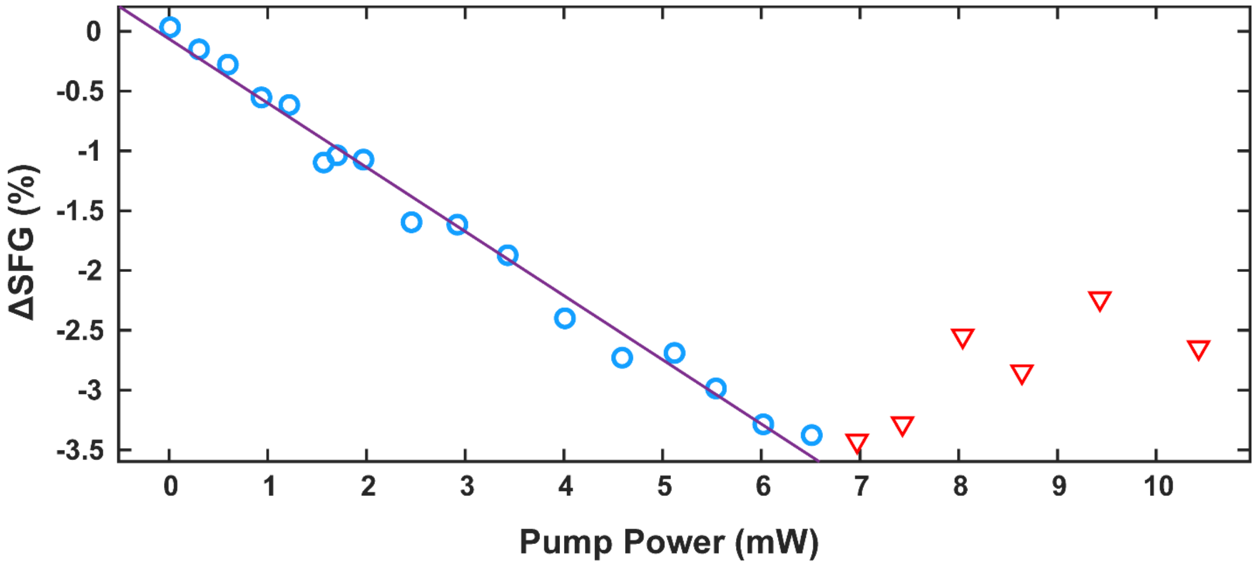

Figure R1. Power dependence on Tr-VSFG dynamics.

4. Did the authors use different polarization to do the experiments? Which polarization was used for the demonstrated results?

Reply: We thank the Reviewer for this question. The polarization dependence result was included in Figure S5b in our original SI. When changing the pump pulse into S polarized, the transient SFG signal become much smaller, suggesting that the direct charge transfer is driven mostly by a dipolar process. For the SFG probe polarization, it was is set as PPP polarization. Other SFG polarization combination, such as SSP, lead to very small signal. The S-pump, PPP probe data showed negligible signal, suggesting that the charge transfer is mostly dipolar allowed process.

Reviewer: 2

Recommendation: This paper may be publishable, but major revision is needed; I would like to be invited to review any future revision.

Comments:

The manuscript by Wang et al. examines the existence of direct interfacial charge transfer (CT) in an all-polymer donor-acceptor heterojunction via transient VSFG and photoelectron spectroscopy. Direct CT could potentially improve the performance of organic electronic devices without using dopants, but it has only been observed in one previous study, and its dynamics and mechanism are largely unknown. Hence,

this study is an important step towards a better understanding of CT in organic semiconductors. I would support its publication after the authors addressed the following comments:

Q1. It would be better to clarify the following terms used in the manuscript: multi-step CT, direct CT, (spontaneous) ground-state CT, photo-induced direct interfacial CT, and indirect CT. It's helpful for the readers to know the differences between them. I would suggest the authors add "photo-induced" to the title to make things clear.

Reply: We recognize that the language used could generate some confusion. Multi-step CT is charge transfer through multiple steps (exciton generation, exciton migration, charge transfer and charge separation etc.), which is also the indirect CT. Direct CT and photo-induced direct interfacial CT in this context refer to the same process. That is photoexcited direct charge transfer from donor to acceptor molecules at interfaces. Ground-state CT is the charge transfer pathway without going through the excited electronic states. We have integrated and clarified all the terms used in the manuscript.

Q2. Line 27-32 on page 6: "Therefore, the donor and acceptor had sufficient orbital mixing for indirect interfacial charge transfer to occur, a prerequisite for direct charge transfer." I don't understand this conclusion. Why is orbital mixing for indirect CT a prerequisite for direct CT?

Reply: We thank the reviewer's question. This statement is based on our previous observation that when there are substantial hybridization between donor and acceptors, direct charge transfer can occur (V.O. Özçelik, Y. Li, W. Xiong, F. Paesani, "Modeling Spontaneous Charge Transfer at Metal/Organic Hybrid Heterostructures", J. Phys. Chem. C, 2020, 124, 8, 4802-4809). We have rewrite the statement in the revision as "Therefore, the donor and acceptor hav sufficient coupling for indirect interfacial charge transfer. The same coupling could facilitate direct charge transfer as well."

Q3. The energy level diagram in Fig. 1 is different from that in Ref. 21, although both looked at P3HT/BBL. The energy gap between P3HT HOMO and BBL LUMO is 1.2 eV in this work but is about 0.6 eV in Ref. 21. Any explanation for this difference?

Reply: We thank for the reviewer's question. It confused us as well. We constructed the band structure based on the details of UPS measurement from Ref.22 (previously 22) supplementary information. We found after the reconstruction, the results from the two works are very similar. We present the detail here. Supplementary Figure 1. showed the ionization energy (energy from HOMO to vacuum level) of BBL equals to 6.33±0.05 eV. LUMO level of BBL related to its own vacuum level (4.15±0.05eV) could be obtained by LEIPS in Supplementary Figure 2(b). Similarly, ionization energy of P3HT could be found in Supplementary Figure 3 note as 4.72eV. Both ionization energies of these two polymers described the energy between of their own HOMO and vacuum level respectively. However, since the vacuum level has a shift between BBL and P3HT for Δ =0.44±0.05eV (obtained from secondary electron cut-off), when calculating the band gap between LUMO of BBL and HOMO of P3HT needs to plus the vacuum level shift:

$$E_g = HOMO(P3HT) - LUMO(BBL) + \Delta = 4.72eV - 4.15eV + 0.44eV = 1.01eV$$

For our result, the band structure of BBL and P3HT are very similar to Ref.22. showing in the table 1 for comparison.

	HOMO(BBL)	LUMO(BBL)	HOMO(P3HT)	LUMO(P3HT)	Vac shift	Eg
Ref 21.	6.33eV	4.15eV	4.72eV	2.72eV	0.44eV	1.01eV
Our results	6.28eV	4.10eV	4.68eV	2.68eV	0.62eV	1.20eV

table. 1. Band information comparison between Ref.22 and our result. All the information of HOMO and LUMO are relative to its own vacuum

In other words, the 0.57 eV gap in Ref.22. was calculated without considering vacuum level shift, and when taking into the vacuum shift, the results from the two publications are similar. The only major difference is the vacuum level shift. The secondary electron cut-off of our P3HT/BBL is 17.30±0.05eV and 17.24±0.05eV for Ref.22. Similarly, the secondary electron cut-off of our BBL is 16.70±0.05eV and 16.80±0.05eV. Therefore, for our vacuum level shift:

$$\Delta = 17.30 \pm 0.05 - 16.68eV \pm 0.05 = 0.62 \pm 0.1eV$$

For Ref.22:

$$\Delta = 17.24 \pm 0.05 - 16.80eV \pm 0.05 = 0.44 \pm 0.1eV$$

The difference between vacuum level shift could be considered into a reasonable instrument error. We have included this comparison in the new SI.

Q4. I wonder if it is possible to estimate the fractions of the photo-induced direct CT pairs that separate into free charges, undergo germinate and non-germinate recombination's (i.e., processes (2), (3), and (4) in Fig. 4(d)) from their transient VSFG.

Reply: We thank the Reviewer for the insightful question. We can use transient VSFG intensity to determine the incident photon to separated charge conversion efficiency. The number of free charge carreirs across interfaces is proportional to the strength of interfacial electric field, on which the transient VSFG intensity is linearly depended, based on the equation below (assuming P3TH/BBL as a parallel capacitor),

$$E = \frac{1}{\varepsilon_r \varepsilon_0} \cdot \frac{Q * e}{A}$$

Where Q is the total number of photoinduced charge at interface, A is irradiative area of 1030nm pump beam (0.0249mm^{-2}), ε_r is dielectric constant of P3HT/BBL system (about 2.5), ε_0 is the permittivity of vacuum (8. 854187817×10^{-12} C/(V*m)), and e is 1.6×10^{-19} C/electron.

When the pump fluence is 2.5mW, the ΔSFG is about 2.5%. Based on Massari's work (ref 27), the same amount SFG modulation was observed at an external potential U of 0.66V, across a layer of 150 nm thickness. Thus, the corresponding interfacial electric field E=U/d= $4.4 \times 10^6\ V/m$.

The charge density (Q/A) separated at interface is given by:

$$Charge\ density = \frac{Q}{A} = E \cdot \varepsilon_r \varepsilon_0 / e = \frac{U}{d} \cdot \varepsilon_r \varepsilon_0 / e = 6.08 \times 10^{14}\ m^{-2}$$

Incident photon flux is calculated based on the measured pump (0.22 μJ) and photon energy (1.2 eV)

$$Photon\ flux = \frac{pump\ energy}{photon\ energy} \cdot \frac{1}{A} = \frac{0.25 \times 10^{-6} J}{1.92 \times 10^{-19} J} \cdot \frac{1}{0.0249\ mm^2} = 5.23 \times 10^{19}\ m^{-2}$$

Therefore, the conversion efficiency from incident photon flux to free charge carrier density is:

$$\eta = 1.2 \times 10^{-5}$$

To estimate the portions that undergo germinate and non-germinate recombination, we will need to know the total number of photons being absorbed, which can be calculated from the absorption cross-section of the planar junction sample. Unfortunately, this quantity is too low to be measured from UV-Vis (only the one of the BHJ can be determined but it has much larger surface areas). We have included the free charge carrier and incident photon flux to free charge carrier conversion efficiency calculation in the SI S7.

Q5. There are some typos in the manuscript (not an exhaustive list): for example, line 15 on page 2, should be "the existence of"; line 22 on page 4, should be "photo-induced direct interfacial charge transfer"

Reply: we thank the Reviewer for pointing out the typo issue. We have corrected all the typos in manuscript.

Reviewer: 3

Comments:

This work characterized direct interfacial charge transfer at the heterojunction between two polymer materials. The charge transfer discovered in wet-processed polymer donor/acceptor interface will provide new platform for both fundamental study and device applications of organic photovoltaics. I would suggest publication in the journal of physical chemistry letters, after addressing the following few questions:

Q1. Would there be any PL signature that may correspond to the radiative recombination of the separated charges at the interface, especially at around 1000 nm similar as the absorption?

Reply: We thank the Reviewer for mentioning the PL signature. In principle, a dipolar absorption should also have a dipolar-allowed emission, and that we should expect to see a PL signal on the red side of 1000 nm. In order to probe any PL signature, we performed the experiment again with more sensitive instrument (Figure R2). The result agreed with the previous measurement in manuscript. The PL quenching indicates charge transfer occurs at donor and acceptor layer. There is extra Raman peak at 880nm, which might be related to interaction between these two layers. However, because the detector's response range is up to 950nm, as it is a Si based detector (SILVER-Nova Super Range TE Cooled Spectrometer), we could not observe any PL near 1000 nm. In the previous plot, there was a label typo

mistake on x-axis of PL spectrum when the figure was prepared in Adobe Illustrator. The detector's response range is shorter 1100 nm. We sincerely apologize for this oversight. We have examined all of our original data and figures to make sure that there are no similar mistakes in other figures. We have included the original plot from OriginLab. here (Figure R2b) and the original data as a supplementary information for review only for the reviewers to examine. We have also uploaded all original data and the code to process the data to a data repository (link here https://github.com/EthanJing1/Data_ChargeTransfer.git) to enhance data transparency. In conclusion, both spectra don't reveal any PL signature below 1000nm. Otherwise, we are not able to conclude if there is any PL signature above 1000nm.

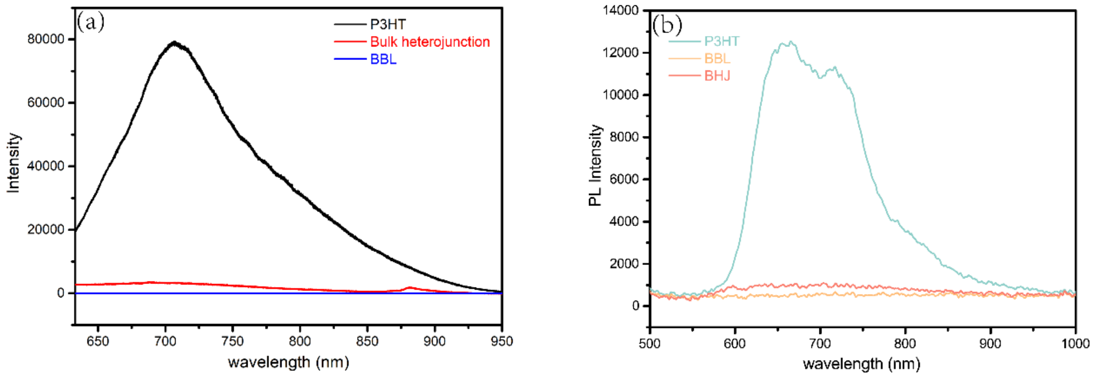

Figure R2. Photoluminescence spectra of bulk heterojunction, pristine P3HT and BBL thin film measured (a) by 515nm laser-based PL instrument. (b) by low energy 515nm LED-based PL instrument.

Q2. What is the polarization of the lasers used for SFG pump-probe experiment? Is it mostly in-plane or out-of-plane?

Reply: We thank the Reviewer's question. The polarization dependence result was included in Figure S5 in our original SI. When changing the pump pulse into S polarized, the transient SFG signal become much smaller, suggesting that the direct charge transfer is driven mostly by a dipolar process. For the SFG probe polarization, it was set as PPP polarization. Other SFG polarization combination, such as SSP, lead to very small signal. The S-pump, PPP probe data showed negligible signal, suggesting that the charge transfer is mostly dipolar allowed process.

Q3. Are there any current or past time-resolved PL or transient absorption measurements for similar polymer interfaces that shows a comparable lifetime of charge carrier transfer state?

Reply: We thank reviewer for mentioning the time-resolved PL or transient absorption measurements, which we have referred to in the original version of our manuscript. Note that we only scan the dynamics to 6 ps (limited by the stage travel and experimental setup), so the 60 ps lifetime only reflects and agrees with the observation that there is a long dynamic component in the charge transfer process. As for P3HT/non-fullerene system, in Tan et al. work(J. Phys. Chem. C 2019, 123, 10, 5826–5832), polaron generated from P3HT excitons exhibit a decay lifetime around 700ps in the blend. Dong et al. (*J. Am. Chem. Soc.* 2021, 143, 20, 7599−7603) shows that other polymer:nonfullerene CT lifetime is about 0.27ns to 1.94ns. Overall, our results agree with the lower limit of lifetime in literatures reasonably well, and the differences could stem from the short scanning range of our experiments or the different nonfullerene materials.

We have included a new statement in the revised manuscript “the recombination lifetime agree with the lower limit of lifetime in literatures reasonably well, and the differences could stem from the short scanning range of our experiments or the different nonfullerene materials” and cited the above literatures

Q4. Could the rate of initial fast charge transfer resolved in the SFG dynamics at times close to time zero?

Reply: We thank the reviewer for the question. The initial charge transfer is likely faster than the instrumental response, so it cannot be resolved, which indicate the direct CT is a vertical excitation. We added a statement in the revision “The rise dynamics of the direct CT is beyond the instrumental response ”.

Q5. What is the polymer thickness prepared from the spin coating process? What would happen if the solvent flow in the spin coating process mix the two polymers, leading to randomly oriented molecules at the interfaces? In that case, would there still be an SFG response if they are homogeneously mixed? Maybe a cross section characterization of the polymer interface would be helpful to illustrate a clear planar heterojunction instead of random mixing.

Reply: We thank reviewer for the suggestion. First, the donor and acceptor have different thickness from spin coating process. The donor BBL layer is about 20nm, whereas the acceptor P3HT layer is about 50nm. Bilayer films were prepared by spin coating from orthogonal solvents, where the chloroform is not miscible with Methanesulfonic acid. This results in a two-dimensional interface between the donor and acceptor polymers. The reference 21 also confirmed that the bilayer structure through annular dark-field STEM image and EDX mapping. To further confirm the planar junction geometry, we conducted SEM measurement (Figure R3a) and the P3HT and BBL layers can be distinguished, suggesting planar junction formation.

We have used mixed solvent to prepare the bulk heterojunction (BHJ) too, which were used to measure UV-Vis spectra of Figure 1b. The SEM cross section image from our own lab confirmed that in BHJ, the donors and acceptors are mixed in one-layer as we only see one layer in bulk heterojunction in Figure R3b. When there are randomly oriented molecules at the interfaces, such as BHJ, there is no SFG response. (Figure R3c) This result can be understood as in BHJ, even charge transfer occurs and generate an DC electric field, this DC field is isotropic, so the net DC field is still zero, which will not generate additional SFG signal through the E-field induced $\chi^{(3)}$ SFG process. We have included this discussion in the new SI.

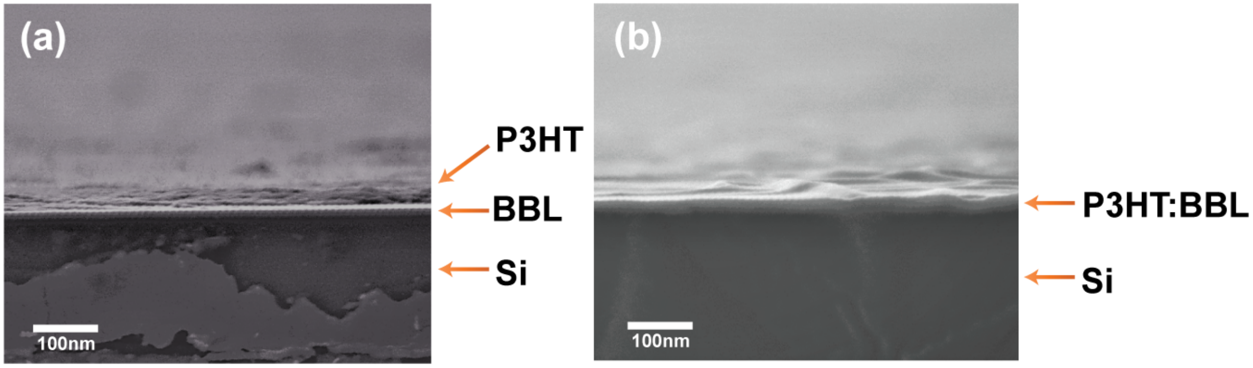

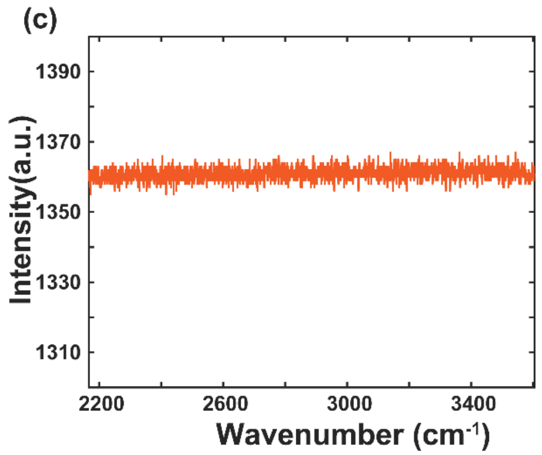

Figure R3. SEM cross section image showing the P3HT/BBL bilayer spin-coated on Si wafer (c) SFG response on BHJ sample

Q6. The UPS measurement of the orbital energies is a crucial information in determining the HOMO LUMO offset. It will be helpful to include the UPS data in a main text figure instead of the supplementary.

Reply: We thank the Reviewer for the suggestion. We have included the UPS data in a main text figure.

Q7. Would there be any expansions for the negative sign of VSFG of component 3 and positive sign of component 2?

Reply: We thank the Reviewer for the insight question. The component 3 comes from the electric field induced $\chi^{(3)}$ response and its negative sign indicates that its phase is opposite from the $\chi^{(2)}$ of the planar junction before charge transfer occurs. The origin of component 2 is less explored. It is likely due to a resonant enhancement from the CT exciton formation, which remain to be further explored experimentally and theoretically.
